# A Ministry of Health

**Published:** 1918-11-02

**Authors:** 


### A Ministry of Health.

At a recent meeting of the Council of the Royal Sanitary Institute the progress made with regard to the Bill for the establishment of a Ministry of Health was under consideration, and the following resolution was passed:—

"The health of the people being of paramount importance in the progress of the nation, the Council of the Royal Sanitary Institute have noted with great satisfaction the progress that has been made and the valuable work accomplished during the past fifty years by the various departments dealing with public health.

"The rapid development under present conditions of the many subsidiary factors affecting the question, and the complexity of the interests involved, make it essential, for the effective continuance and development of the work, that so far as possible all matters relating to public health should be co-ordinated in one department as a Ministry of Health.

"The Council therefore desire to urge that the matter is one of pressing public importance, and trust that it may receive the early attention of His Majesty's Government."

